# Supplementary material for: Impacts of Dietary Protein and Niacin Deficiency on Reproduction Performance, Body Growth, and Gut Microbiota of Female Hamsters (Tscherskia triton) and Their Offspring
Source: Microbiol Spectr. 2022 Nov 1;10(6):e00157-22. doi: 10.1128/spectrum.00157-22 (PMC9784777; doi:10.1128/spectrum.00157-22)
Supplement: Supplemental file 1 — Supplemental material. Download spectrum.00157-22-s0001.pdf, PDF file, 0.4 MB [file spectrum.00157-22-s0001.pdf]

Supplementary Materials for

Manuscript: *"Impacts of dietary protein and niacin deficiency on reproduction performance, body growth and gut microbiota of female hamsters (Tscherskia triton) and their offspring*"

This file includes:

Supplementary Table S1 to S10

Table S1. Sample size of female (maternal) hamsters under four dietary treatments (2 × 2-factor design) in different experimental periods.

Group	Adaptation stage	Cohabitation test	Mating	Pregnancy	Number of litters	Infanticide
Normal protein & Niacin^{+}	11(**1**)	10(**1**)	8	5	5	0
Normal protein & Niacin^{-}	12(**2**)	10	10	6	6	1
Low protein & Niacin^{+}	14(**2**)	12(**1**)	7	5	5	0
Low protein & Niacin^{-}	14(**1**)	13(**1**)	10	3	3	0

Bold numbers in brackets represent the number of hamsters died or injured in the process of the experiment. In the cohabitation test, a total of 7 females rejected to mate with any males within 14 days. One female hamster with 4 (2 male and 2 female) pups displayed infanticide behavior two weeks after parturition, and was excluded for further analysis. A total of 68 pups from four diet treatment groups (5, 5, 5, and 3 litters from Normal protein & Niacin+, Normal protein & Niacin-, Low-protein & Niacin+, and Low-protein & Niacin-, respectively) were sampled for fecal microbiota analysis.

Table S2. Effects of protein and niacin diets on body weight of female hamsters and offspring hamsters by using Linear mix effect model or two-way anova methods.

**Female hamster (LMM)**	*F*	*p*-value
Protein	2.56	0.116
Niacin	0.82	0.371
Time	1.11	0.293
Protein × Time	25.41	**<0.0001**
Niacin × Time	2.20	0.139
Protein × Niacin	0.14	0.708
**Offspring hamster (anova)**		
Protein	130.25	**<0.0001**
Niacin	0.13	0.722
Protein × Niacin	0.15	0.700

Table S3. Effects of protein and niacin diets on reproduction performance of female hamsters by using Wald Chisquare test of GLM (Generalized linear model).

	Distribution family	Variables	Statistic	*p*-value	R^2
Number of cohabitation tests	Poisson (link: log)	Protein	13.50	**<0.001**	0.350
		Niacin	0.25	0.617	
		Protein × Niacin	2.46	0.117	
Mount latency (s)	Poisson (link: log)	Protein	8.04	**<0.01**	0.009
		Niacin	2.60	0.107	
		Protein × Niacin	0.01	0.917	
Mount frequency	Poisson (link: log)	Protein	0.60	0.437	0.040
		Niacin	0.31	0.579	
		Protein × Niacin	0.0025	0.960	
Litter size	Gaussian (link: identity)	Protein	17.033	**<0.0001**	0.541
		Niacin	1.50	0.220	
		Protein × Niacin	0.27	0.600	
Sex ratio	Binomial (link: logit)	Protein	0.76	0.382	0.026
		Niacin	1.34	0.248	
		Protein × Niacin	0.01	0.932	

Effect sizes of GLM were presented as R^2.

Table S4. Effects of protein and niacin diets on alpha diversity of gut microbiota of female and offspring hamsters by using two-way ancova.

index	term	Female hamsters		Offspring hamsters	
		F	*p* value	*F*	*p* value
Shannon	Protein	1.77	0.192	7.83	**0.007**
	Niacin	5.66	**0.024**	4.82	**0.032**
	Library size	55.87	**<0.001**	35.09	**<0.001**
Chao1	Protein	0.58	0.453	14.03	**<0.001**
	Niacin	0.61	0.441	5.14	**0.027**
	Library size	17.34	**<0.001**	0.17	0.683
Observed_features	Protein	0.37	0.547	11.12	**0.0014**
	Niacin	0.79	0.381	5.05	**0.028**
	Library size	17.09	**<0.001**	0.46	0.499
Phylogenetic diversity	Protein	0.14	0.711	0.46	0.500
	Niacin	0.10	0.751	0.37	0.543
	Library size	5.36	**0.027**	0.70	0.407

Table S5. Summary of differential microbiota at genus level in LPD diet groups for female hamsters.

Phylum	Genus	beta	W	*q* value	95% CI
Firmicutes	*Allobaculum*	0.13	-0.30	<0.0001	(-0.99, 0.73)
Firmicutes	*Bacteroides [Ruminococcaceae]*	-1.66	-3.87	<0.05	(-2.50. -0.82)
Firmicutes	*Gemmiger[Ruminococcaceae]*	0.38	1.09	<0.0001	(-0.31, 1.07)
Firmicutes	*Oscillospira[Ruminococcaceae]*	0.60	1.32	<0.0001	(-0.29. 1.49)
Firmicutes	*Unclassified Bacillaceae*	-0.91	-1.76	<0.0001	(-1.93, 0.11)
Firmicutes	*Unclassified Firmicutes*	2.07	3.66	<0.05	(0.96, 3.18)
Firmicutes	*Unclassified Peptostreptococcaceae*	0.09	0.33	<0.0001	(-0.46, 0.65)
Firmicutes	*Unclassified Veillonellaceae*	-0.27	-0.49	<0.0001	(-1.33, 0.80)
Bacteroidetes	*Dysgonomonas*	-0.26	-1.25	<0.0001	(-0.67, 0.15)
Bacteroidetes	*Elizabethkingia*	0.43	1.52	<0.0001	(-0.13, 0.98)
Bacteroidetes	*Macellibacteroides*	0.30	0.83	<0.0001	(-0.41, 1.02)
Bacteroidetes	*Unclassified [Paraprevotellaceae]*	0.54	0.85	<0.0001	(-0.70, 1.78)
Bacteroidetes	*Unclassified [Weeksellaceae]*	0.62	1.92	<0.0001	(-0.01, 1.26)
Bacteroidetes	*Unclassified Bacteroidetes*	2.00	3.37	<0.0001	(0.84, 3.16)
Bacteroidetes	*YRC22*	-2.33	-3.19	<0.0001	(-3.77, -0.90)
Proteobacteria	*Acinetobacter*	1.02	2.38	<0.0001	(0.18, 1.87)
Proteobacteria	*Georgfuchsia*	-0.77	-1.49	<0.0001	(-1.78, 0.24)
Proteobacteria	*Oleomonas*	0.55	2.06	<0.0001	(0.03, 1.06)
Proteobacteria	*Unclassified Gammaproteobacteria*	0.40	1.14	<0.0001	(-0.29, 1.10)
Proteobacteria	*Unclassified Proteobacteria*	-0.08	-0.17	<0.0001	(-0.95, 0.80)
Spirochaetes	*Spirochaeta*	0.99	2.99	<0.0001	(0.34, 1.64)
Actinobacteria	*Corynebacterium*	0.26	0.78	<0.0001	(-0.39, 0.90)
Actinobacteria	*Millisia*	1.73	4.79	<0.0001	(1.02, 2.44)
Tenericutes	*Anaeroplasma*	-0.81	-1.58	<0.0001	(-1.83, 0.20)
Tenericutes	*Mycoplasma*	0.74	1.28	<0.0001	(-0.39, 1.88)
Deferribacteres	*Mucispirillum*	0.44	0.97	<0.0001	(-0.45, 1.32)
Fusobacteria	*Unclassified Fusobacteriaceae*	-0.52	-0.61	<0.0001	(-2.18, 1.15)

A positive or negative W-value indicates a decrease or increase in absolute abundance in low protein diet, respectively. *p*-values were adjusted using “holm” correction and presented as *q*-values.

Table S6. Summary of differential microbiota at genus level in offspring hamsters under maternal LPD diet.

Phylum	Genus	beta	W	*q* value	95% CI
Firmicutes	*Butyricicoccus*	0.84	2.56	<0.0001	(0.20, 1.49)
Firmicutes	*Christensenella*	-0.04	-0.22	<0.0001	(-0.40, 0.32)
Firmicutes	*Clostridium [Erysipelotrichaceae]*	-0.26	-0.95	<0.0001	(-0.78, 0.27)
Firmicutes	*Dorea*	2.31	5.95	<0.0001	(1.55, 3.07)
Firmicutes	*Gemmiger*	0.21	0.81	<0.0001	(-0.29, 0.71)
Firmicutes	*Lachnospira*	0.32	1.01	<0.0001	(-0.30, 0.95)
Firmicutes	*Lactobacillus*	-1.06	-7.22	<0.0001	(-1.35, -0.77)
Firmicutes	*Proteiniborus*	0.32	1.34	<0.0001	(-0.15, 0.78)
Firmicutes	*Ruminococcus[Ruminococcaceae]*	1.93	3.95	<0.0001	(0.97, 2.89)
Firmicutes	*Ruminococcus[Lachnospiraceae]*	0.61	1.37	<0.0001	(-0.26, 1.47)
Firmicutes	*Sporobacter*	2.22	5.19	<0.0001	(1.38, 3.05)
Firmicutes	*Unclassified Firmicutes*	1.44	4.18	<0.01	(0.77, 2.12)
Firmicutes	*Unclassified Lachnospiraceae*	1.50	7.33	<0.0001	(1.10, 1.90)
Firmicutes	*Unclassified Lactobacillaceae*	-1.41	-6.81	<0.0001	(-1.81, -1.00)
Firmicutes	*Unclassified Peptostreptococcaceae*	-0.33	-1.23	<0.0001	(-0.87, 0.20)
Bacteroidetes	*Elizabethkingia*	-1.29	-4.26	<0.0001	(-1.88, -0.69)
Bacteroidetes	*Unclassified [Paraprevotellaceae]*	-0.88	-2.98	<0.0001	(-1.44, -0.30)
Bacteroidetes	*Unclassified Rikenellaceae*	-0.24	-1.22	<0.0001	(-0.61, 0.14)
Bacteroidetes	*Unclassified S24-7*	-0.67	-5.16	<0.0001	(-0.91, -0.41)
Proteobacteria	*Alkanindiges*	-1.37	-3.80	<0.0001	(-2.08, -0.66)
Proteobacteria	*Bordetella*	-1.82	-5.15	<0.0001	(-2.51, -1.12)
Proteobacteria	*Desulfovibrio*	2.56	5.84	<0.0001	(1.70, 3.42)
Proteobacteria	*Helicobacter*	2.07	5.28	<0.0001	(1.30, 2.84)
Proteobacteria	*Nitrobacteria*	-0.29	-1.27	<0.0001	(-0.74, 0.16)
Proteobacteria	*Pseudomonas*	-2.67	-5.40	<0.0001	(-3.64, -1.70)
Proteobacteria	*Pseudoxanthomonas*	-1.78	-4.94	<0.0001	(-2.49, -1.08)
Proteobacteria	*Unclassified Comamonadaceae*	-1.57	-3.81	<0.0001	(-2.37, -0.76)
Proteobacteria	*Unclassified Helicobacteraceae*	1.85	3.35	<0.05	(0.77, 2.93)
Proteobacteria	*Unclassified Proteobacteria*	1.48	3.63	<0.05	(0.68, 2.28)
Spirochaetes	*Brachyspira*	0.11	0.38	<0.0001	(-0.45, 0.67)
Spirochaetes	*Spirochaeta*	0.06	0.34	<0.0001	(-0.30, 0.42)
Spirochaetes	*Treponema*	2.63	4.29	<0.01	(1.43, 3.83)
Actinobacteria	*Arthrobacter*	-0.44	-1.80	<0.0001	(-0.91, 0.04)
Actinobacteria	*Bifidobacterium*	-1.98	-4.32	<0.01	(-2.89, -1.08)
Actinobacteria	*Millisia*	-1.60	-4.03	<0.0001	(-2.38, -0.82)
Actinobacteria	*Olsenella*	-1.91	-3.93	<0.01	(-2.86, -0.96)
Actinobacteria	*Unclassified Actinomycetales*	-1.40	-3.79	<0.0001	(-2.13, -0.68)
Actinobacteria	*Unclassified Coriobacteriaceae*	-2.02	-6.79	<0.0001	(-2.61, -1.44)
Tenericutes	*Anaeroplasma*	-0.01	-0.03	<0.0001	(-0.72, 0.69)
Cyanobacteria	*Unclassified YS2*	-1.56	-3.70	<0.05	(-2.38, -0.73)

Phylum	Genus	beta	W	*q* value	95% CI
Deferribacteres	*Mucispirillum*	-0.00	-0.01	<0.0001	(-0.42, 0.41)

A positive or negative W-value indicates a decrease or increase in absolute abundance in low protein diet, respectively. *p*-values were adjusted using “holm” correction and presented as *q*-values.

Table S7. Summary of differential microbiota at genus level in offspring of maternal Niacin^{-} diet.

Phylum	Genus	beta	W	*q*-value	95% CI
Firmicutes	*Clostridium [Erysipelotrichaceae]*	.69	3.17	<0.0001	(0.26, 1.12)
Firmicutes	*Hespellia*	0.39	1.39	<0.0001	(-0.16, 0.95)
Firmicutes	*Macrococcus*	0.63	3.71	<0.0001	(0.30, 0.97)
Firmicutes	*Proteiniborus*	-0.75	-2.54	<0.0001	(-1.34, -0.17)
Bacteroidetes	*Unclassified [Paraprevotellaceae]*	0.57	3.03	<0.0001	(0.20, 0.94)
Bacteroidetes	*Unclassified Porphyromonadaceae*	0.52	2.81	<0.0001	(0.16, 0.89)
Proteobacteria	*Alkanindiges*	0.49	1.94	<0.0001	(-0.00, 0.98)
Proteobacteria	*Unclassified Comamonadaceae*	0.52	1.80	<0.0001	(-0.05, 1.08)
Spirochaetes	*Spirochaeta*	-0.21	-1.06	<0.0001	(-0.59, 0.17)
Actinobacteria	*Arthrobacter*	0.41	2.42	<0.0001	(0.07, 0.74)
Actinobacteria	*Unclassified Actinomycetales*	-0.08	-0.27	<0.0001	(-0.65, 0.50)
Deferribacteres	*Mucispirillum*	0.71	3.70	<0.0001	(0.33, 1.08)

A positive or negative W-value indicates a decrease or increase in absolute abundance in low protein diet, respectively. *p*-values were adjusted using “holm” correction and presented as *q*-values.

Table S8. Effects of protein and niacin diets on interindividual variation and composition of KO functional orthologs and KEGG pathways.

		Female hamster			Offspring hamster		
**BETADISPER**		***F***	***p***		***F***	***p***	
Functional orthologs	Protein (NPD vs. LPD)	0.10	0.723		0.64	0.425	
	Niacin (Niacin$^+$ vs. Niacin$^-$)	0.27	0.598		0.15	0.720	
KEGG pathway	Protein (NPD vs. LPD)	0.61	0.452		0.13	0.728	
	Niacin (Niacin$^+$ vs. Niacin$^-$)	0.65	0.417		0.85	0.395	
**PERMANOVA**		**Pseudo-*F***	***p***	***R*²**	**Pseudo-*F***	***p***	***R*²**
Functional orthologs	Protein (NPD vs. LPD)	1.76	0.136	0.049	3.58	**0.021**	0.158
	Niacin (Niacin$^+$ vs. Niacin$^-$)	0.50	0.761	0.014	0.74	0.620	0.036
	Protein × niacin	1.55	0.168	0.043	0.88	0.430	0.037
	Dam's ID	——	——	——	2.28	**0.002**	0.637
KEGG pathway	Protein (NPD vs. LPD)	1.57	0.205	0.043	2.57	0.073	0.086
	Niacin (Niacin$^+$ vs. Niacin$^-$)	0.27	0.880	0.008	1.01	0.385	0.037
	Protein × niacin	2.14	0.085	0.060	0.71	0.488	0.023
	Dam's ID	——	——	——	1.70	**0.027**	0.477

Abbreviations: NPD, normal protein diet; LPD, low-protein diet; Niacin$^+$, niacin supplement diet; Niacin$^-$, niacin deficiency diet.

Table S9. Predicted KEGG pathways that significantly enriched or depleted in offspring hamsters of maternal LPD diet groups.

Pathway	Level3	Level2	Level1	Effect size	LDA score	*p*-value	enriched_group
ko02040	Cellular Processes	Cell motility	Flagellar assembly	3.29	2.88	2.99E-08	NPD
ko02024	Cellular Processes	Cellular community - prokaryotes	Quorum sensing	4.42	2.73	0.003142	NPD
ko02030	Cellular Processes	Cell motility	Bacterial chemotaxis	3.39	2.90	4.10E-07	NPD
ko02010	Environmental Information Processing	Membrane transport	ABC transporters	4.82	3.26	0.001228	NPD
ko02020	Environmental Information Processing	Signal transduction	Two-component system	4.54	3.24	7.85E-05	NPD
ko03010	Genetic Information Processing	Translation	Ribosome	4.31	2.71	0.011022	LPD
ko03018	Genetic Information Processing	Folding, sorting and degradation	RNA degradation	3.77	2.31	0.002119	LPD
ko00970	Genetic Information Processing	Translation	Aminoacyl-tRNA biosynthesis	4.02	2.29	0.047201	LPD
ko05132	Human Diseases	Infectious diseases: Bacterial	Salmonella infection	2.46	2.02	8.74E-07	NPD
ko05230	Human Diseases	Cancers: Overview	Central carbon metabolism in cancer	3.38	2.24	1.22E-04	LPD
ko05150	Human Diseases	Infectious diseases: Bacterial	Staphylococcus aureus infection	3.19	2.02	0.004791	LPD
ko01503	Human Diseases	Drug resistance: Antimicrobial	Cationic antimicrobial peptide (CAMP) resistance	3.54	2.14	5.72E-04	LPD
ko00564	Metabolism	Lipid metabolism	Glycerophospholipid metabolism	3.73	2.03	0.022033	LPD
ko00250	Metabolism	Amino acid metabolism	Alanine, aspartate and glutamate metabolism	3.91	2.06	0.029982	LPD
ko00600	Metabolism	Lipid metabolism	Sphingolipid metabolism	3.23	2.20	0.040281	LPD
ko00300	Metabolism	Amino acid metabolism	Lysine biosynthesis	3.87	2.36	0.006636	LPD
ko00720	Metabolism	Energy metabolism	Carbon fixation pathways in prokaryotes	3.94	2.46	5.44E-04	NPD

Pathway	Level3	Level2	Level1	Effect size	LDA score	*p*-value	enriched_group
ko00740	Metabolism	Metabolism of cofactors and vitamins	Riboflavin metabolism	3.79	2.35	0.040281	LPD
ko01200	Metabolism	Global and overview maps	Carbon metabolism	4.37	2.48	0.005646	NPD
ko00680	Metabolism	Energy metabolism	Methane metabolism	3.81	2.11	5.93E-05	NPD
ko00040	Metabolism	Carbohydrate metabolism	Pentose and glucuronate interconversions	3.44	2.10	0.00542	NPD
ko01040	Metabolism	Lipid metabolism	Biosynthesis of unsaturated fatty acids	3.15	2.03	2.65E-05	LPD
ko01210	Metabolism	Global and overview maps	2-Oxocarboxylic acid metabolism	3.56	2.31	0.012343	NPD
ko00400	Metabolism	Amino acid metabolism	Phenylalanine, tyrosine and tryptophan biosynthesis	3.30	2.25	0.0133	NPD
ko00290	Metabolism	Amino acid metabolism	Valine, leucine and isoleucine biosynthesis	3.04	2.10	0.004409	NPD
ko01220	Metabolism	Global and overview maps	Degradation of aromatic compounds	3.52	2.10	0.031002	LPD
ko00020	Metabolism	Carbohydrate metabolism	Citrate cycle (TCA cycle)	3.66	2.18	0.032051	NPD
ko00340	Metabolism	Amino acid metabolism	Histidine metabolism	2.97	2.20	0.0133	NPD
ko00910	Metabolism	Energy metabolism	Nitrogen metabolism	3.31	2.09	0.007479	NPD
ko00230	Metabolism	Nucleotide metabolism	Purine metabolism	4.34	2.62	0.03424	LPD
ko00240	Metabolism	Nucleotide metabolism	Pyrimidine metabolism	4.24	2.55	0.039005	LPD
ko04922	Organismal Systems	Endocrine system	Glucagon signaling pathway	3.45	2.26	0.002027	LPD

Table S10. Predicted KO functional orthologs that significantly enriched or depleted in offspring hamsters of maternal LPD diet groups.

Orthologs	Description	Effect size	LDA score	*p*-value	enriched_group
K00016	L-lactate dehydrogenase [EC:1.1.1.27]	3.31	2.11	0.009097319	LPD
K00571	site-specific DNA-methyltransferase (adenine-specific) [EC:2.1.1.72]	3.13	2.14	0.000159046	NPD
K00615	transketolase [EC:2.2.1.1]	2.63	2.04	2.81E-05	NPD
K01104	protein-tyrosine phosphatase [EC:3.1.3.48]	3.43	2.24	0.001410768	LPD
K01372	bleomycin hydrolase [EC:3.4.22.40]	3.27	2.05	0.019134937	LPD
K01448	N-acetylmuramoyl-L-alanine amidase [EC:3.5.1.28]	2.53	2.09	2.08E-06	NPD
K02010	iron (III) transport system ATP-binding protein [EC:3.6.3.30]	3.34	2.04	0.000544358	NPD
K02025	multiple sugar transport system permease protein	3.21	2.42	0.000142925	NPD
K02026	multiple sugar transport system permease protein	3.20	2.47	5.46E-06	NPD
K02027	multiple sugar transport system substrate-binding protein	3.14	2.24	0.000971571	NPD
K02029	polar amino acid transport system permease protein	3.64	2.23	0.003009383	LPD
K02030	polar amino acid transport system substrate-binding protein	3.47	2.16	0.002420163	LPD
K02031	peptide/nickel transport system ATP-binding protein	3.72	2.35	0.013300006	NPD
K02032	peptide/nickel transport system ATP-binding protein	3.66	2.48	1.56E-05	NPD
K02035	peptide/nickel transport system substrate-binding protein	3.14	2.04	0.000493112	NPD
K02056	simple sugar transport system ATP-binding protein [EC:3.6.3.17]	3.08	2.34	1.52E-07	NPD
K02057	simple sugar transport system permease protein	2.90	2.27	7.01E-06	NPD
K02071	D-methionine transport system ATP-binding protein	3.55	2.13	0.002759546	LPD
K02392	flagellar basal-body rod protein FlgG	2.39	2.01	5.41E-09	NPD
K02483	two-component system, OmpR family, response regulator	3.56	2.16	0.04293811	NPD
K03088	RNA polymerase sigma-70 factor, ECF subfamily	2.97	2.41	0.000729415	NPD
K03205	type IV secretion system protein VirD4	2.69	2.31	4.05E-08	NPD
K03293	amino acid transporter, AAT family	3.48	2.29	0.005645941	LPD

Orthologs	Description	Effect size	LDA score	*p*-value	enriched_group
K03294	basic amino acid/polyamine antiporter, APA family	3.42	2.22	0.004054904	LPD
K03406	methyl-accepting chemotaxis protein	3.08	2.67	2.18E-07	NPD
K03446	MFS transporter, DHA2 family, multidrug resistance protein	3.14	2.00	0.009456313	LPD
K03497	chromosome partitioning protein, ParB family	3.14	2.04	8.74E-07	NPD
K03696	ATP-dependent Clp protease ATP-binding subunit ClpC	3.30	2.02	0.00046921	LPD
K06158	ATP-binding cassette, subfamily F, member 3	3.49	2.03	0.018464671	LPD
K06221	2,5-diketo-D-gluconate reductase A [EC:1.1.1.346]	3.34	2.18	0.012813626	LPD
K06222	2,5-diketo-D-gluconate reductase B [EC:1.1.1.346]	3.01	2.04	0.001771606	LPD
K06223	DNA adenine methylase [EC:2.1.1.72]	2.58	2.10	6.28E-05	NPD
K06400	site-specific DNA recombinase	2.64	2.03	3.97E-06	NPD
K06871	uncharacterized protein	2.41	2.06	4.20E-08	NPD
K06889	uncharacterized protein	3.26	2.11	6.59E-06	LPD
K07482	transposase, IS30 family	3.34	2.27	0.022033204	LPD
K07487	transposase	3.41	2.34	0.02053953	LPD
K07496	putative transposase	3.53	2.37	0.012343053	LPD
K07718	two-component system, sensor histidine kinase YesM [EC:2.7.13.3]	2.68	2.37	9.49E-08	NPD
K07720	two-component system, response regulator YesN	3.12	2.48	2.03E-07	NPD
K07814	putative two-component system response regulator	2.54	2.09	2.65E-05	NPD
K08659	dipeptidase [EC:3.4.-.-]	3.46	2.22	0.035382237	LPD
K09810	lipoprotein-releasing system ATP-binding protein [EC:3.6.3.-]	2.72	2.12	0.001771606	NPD
K10119	raffinose/stachyose/melibiose transport system permease protein	3.03	2.04	0.008415625	NPD
K10439	ribose transport system substrate-binding protein	2.51	2.18	2.85E-09	NPD
K10440	ribose transport system permease protein	2.56	2.23	1.19E-08	NPD
K10441	ribose transport system ATP-binding protein [EC:3.6.3.17]	3.02	2.32	3.16E-05	NPD

Orthologs	Description	Effect size	LDA score	*p*-value	enriched_group
K11733	lysine-specific permease	3.24	2.09	0.002641868	LPD
K11907	type VI secretion system protein VasG	3.24	2.03	0.000176867	LPD
K12132	eukaryotic-like serine/threonine-protein kinase [EC:2.7.11.1]	2.93	2.10	1.56E-05	LPD
K17319	putative aldouronate transport system permease protein	2.41	2.09	1.52E-07	NPD
K17320	putative aldouronate transport system permease protein	2.45	2.10	9.35E-07	NPD
K18926	MFS transporter, DHA2 family, lincomycin resistance protein	3.27	2.14	0.013300006	LPD
